# Tetherin antagonism by HIV-1 and its SIV precursors

**DOI:** 10.1186/1742-4690-8-S2-O8

**Published:** 2011-10-03

**Authors:** Frank Kirchhoff

**Affiliations:** 1 Institute of Molecular Virology, Ulm University Medical Center, 89081 Ulm, Germany

## 

Retroviruses have evolved effective strategies to evade the host immune response, such as high variability and latent infection. In addition, primate lentiviruses, such as HIV-1, have acquired several «accessory» genes that antagonize antiviral host restriction factors and facilitate viral immune evasion, thereby allowing continuous and efficient viral replication despite apparently strong innate and acquired immune responses. Here, I summarize some of our current knowledge on the function of the viral *vpu* and *nef* genes, with a particular focus on their capability to antagonize an antiviral factor named "tetherin" (BST-2) that tethers nascent virions at the cell surface. Evidence will be presented that switches between Nef- and Vpu-mediated tetherin antagonism preceded the emergence of HIV-1. Furthermore, it will be discussed why tetherin most likely poses a significant hurdle to cross-species transmissions of simian immunodeficiency viruses to humans. To our current knowledge only pandemic HIV-1 M (major) strains mastered this barrier perfectly by,,regaining" efficient tetherin activity by Vpu following the four independent cross-species transmissions that resulted in HIV-1 groups M, O, N, and P. This may potentially explain why HIV-1 M it is almost entirely responsible for the HIV/AIDS pandemic.

